# Documenting museum records of West African Coccinellidae (Coleoptera) in Benin and Senegal

**DOI:** 10.3897/BDJ.8.e47340

**Published:** 2020-01-17

**Authors:** Kwevitoukoui Hounkpati, Joseph V. McHugh, Abdoul Aziz Niang, Georg Goergen

**Affiliations:** 1 Grain de Sel Togo, Inc., Athens, United States of America Grain de Sel Togo, Inc. Athens United States of America; 2 Department of Entomology, University of Georgia, Athens, United States of America Department of Entomology, University of Georgia Athens United States of America; 3 Laboratoire de Zoologie des Invertébrés Terrestres, Institut Fondamental d’Afrique Noire Cheikh Anta Diop, Dakar, Senegal Laboratoire de Zoologie des Invertébrés Terrestres, Institut Fondamental d’Afrique Noire Cheikh Anta Diop Dakar Senegal; 4 International Institute of Tropical Agriculture, Cotonou, Benin International Institute of Tropical Agriculture Cotonou Benin

**Keywords:** Africa, Coccinelloidea, Coccinellinae, distribution, diversity, insect collection, inventory, Microweiseinae, taxonomy, museum, systematics, lady beetles, ladybugs.

## Abstract

**Background:**

This work provides a preliminary inventory of West African Coccinellidae.

This was based on the West African Coccinellidae (WAC) specimens in the holdings of insect collections at the Laboratoire de Zoologie des Invertébrés Terrestres at the Institut Fondamental d’Afrique Noire Cheikh Anta Diop (IFAN), Senegal and the Biodiversity Center at the International Institute of Tropical Agriculture (IITAB), Benin.

**New information:**

A total of 129 species representing 11 tribes and 40 genera is reported, including one species of the subfamily Microweiseinae and 128 species of the subfamily Coccinellinae. The geographic distribution of collection localities is presented for these species. *Cheilomenes lunata* (Fabricius, 1775), *Cheilomenes propinqua* (Mulsant, 1850), *Cheilomenes sulphurea* (Olivier, 1791), *Chnootriba elaterii* (Rossi, 1794), *Chnootriba similis* (Thunberg, 1781), *Exochomus laeviusculus* Weise, 1909, *Hyperaspis delicatula* (Mulsant, 1850) and *Hyperaspis pumila* Mulsant, 1850 are the best represented species in these collections.

## Introduction

Natural history museums play a critical role in science and education (Gropp 2018, Holmes et al. 2016). They contribute fundamental data, necessary for understanding the biodiversity of Earth across temporal and geographic ranges. Biological information derived from specimen data provides essential information supporting a wide range of basic and applied biological endeavours. Museum specimens often play an important role in the recognition of threatened taxa, allowing the scientific community to propose appropriate action (e.g. Thomson et al. 2018, Mikula et al. 2018). Other activities that benefit from specimen-based biological information include the monitoring of environmental change, protecting public health and safety and enhancing agriculture, to name a few (Suarez and Tsutsui 2004).

In agriculture, biological collections can provide vitally important information about pest species, including phenology, food preferences, behaviour, ecological associations etc. (Escalona et al. 2017). For invasive species, museum data can help to identify the point of entry, the date of introduction, the rate of expansion and the native distributional range, which is where natural enemies (potential biocontrol agents) might be found.

The full benefits of natural history museums can only be realised if these collections are known and accessible to the public and scientific communities worldwide, thereby enabling cooperation between local and distant scientists to explore and advance our knowledge of global biodiversity. Unfortunately, access to biological collections is limited for researchers in some regions, like Africa. Much of the available museum material of African origin is housed in natural history museums or private collections in Europe (Klopper et al. 2002, Miller and Rogo 2001). There are some major African national or regional natural history museums. The majority of their holdings comprise subsets of material that was collected by missionaries, explorers and scientists from European institutions (Medler 1980, Scholtz and Mansell 2017). There are some natural history museums in West Africa, but these have remained overlooked by the research community.

Coccinellidae Latreille, 1807, commonly known as ladybugs, are small beetles ranging from 0.8 to 18 mm (Seago et al. 2011). Although some coccinellids are phytophagous or fungivorous (Iperti 1999), nearly 70% of species are considered predaceous, preying on aphids, mealybugs, scale insects, thrips, leaf hoppers, mites and other soft bodied insects (Giorgi et al. 2009, Riddick 2017, Shah and Khan 2014, Ślipiński and Tomaszewska 2010, Szawaryn et al. 2015). One of the earliest and most successful examples of biocontrol was the management of cottony-cushion scale, *Icerya purchasi* Maskell, 1879, on citrus crops using the vedalia lady beetle, *Rodolia cardinalis* (Mulsant, 1850), in California during the 1890s.

Due to their economic importance, major regional taxonomic works have been published for the coccinellid faunas of North America, Europe, Palearctic Region, Russian Far East, other parts of the former USSR, Oriental region, Central Asia, Japan, Vietnam, China and Australia (e.g. Giorgi et al. 2009, Tomaszewska and Szawaryn 2013, Tomaszewska and Ślipiński 2013, Ślipiński et al. 2012). Unfortunately, nothing comparable exists for Africa. The identification of African lady beetles has been done almost exclusively by European entomologists and explorers (Medler 1980). As a result, valuable reference specimens and their associated collection data are often inaccessible to researchers and agencies in Africa (Scholtz and Mansell 2017).

Coccinellidae has been the focus of several recent phylogenetic studies as researchers attempt to understand the evolution of the group and to improve its classification (e.g. Escalona and Ślipiński 2012, Kobayashi et al. 2011, Nedved and Kovář 2012, Robertson et al. 2015, Seago et al. 2011, Tomaszewska and Szawaryn 2013, Tomaszewska and Ślipiński 2013, Ślipiński et al. 2012). Unfortunately, endemic African taxa are very poorly represented in these studies, probably because appropriate material was unavailable and because the state of taxonomy for those groups was not mature.

Although Africa is well known for its rich and charismatic vertebrate diversity, there is far less appreciation for the great diversity of other taxa there. It is estimated that 100,000 species of insects are currently known from the continent and conservative estimates put the total number of insect species there at about 600,000, yet few research collections of insects exist on the continent (Miller and Rogo 2001). While entomological research collections in some African countries (e.g. Algeria, Egypt, Ghana, Kenya, Libya, Malawi, Morocco, Mozambique, South Africa, Tanzania, Uganda and Zambia) have appeared for some time in registries of museums (e.g. Arnett et al. 1993, Evenhuis 2019), others remain virtually unknown to the outside world, especially those in French West African countries (*Afrique Occidentale Française*). This is true for the two largest reference collections of arthropods in West Africa, the Biodiversity Center at the International Institute of Tropical Agriculture (IITAB) in Benin and the Laboratoire de Zoologie des Invertébrés Terrestres at the Institut Fondamental d’Afrique Noire Cheikh Anta Diop (IFAN) in Senegal. These two important resources were absent from compilations of insect and spider collections of the world until just recently when they were added to a web-based listing of biological collections by Evenhuis (2019) following an enquiry by the senior author.

**Figure 1.**
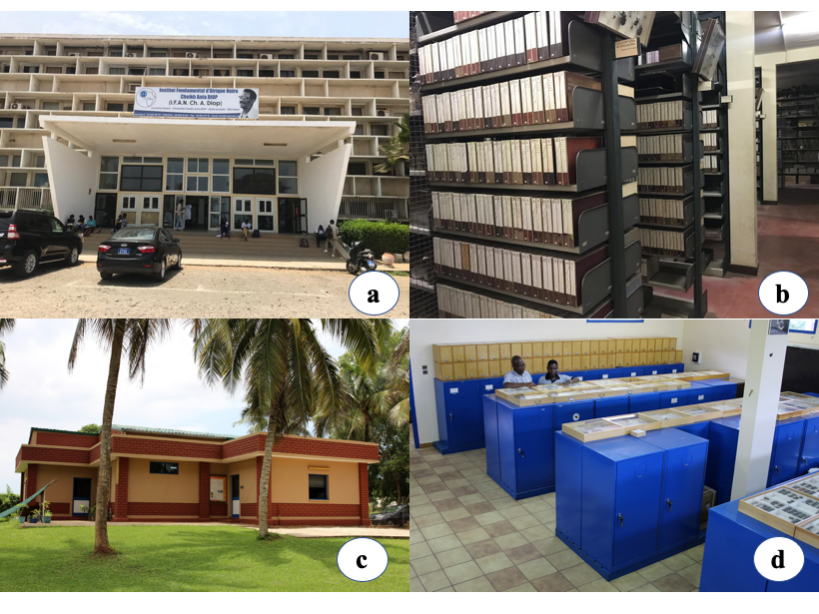
Photos of the two largest West African insect museums: **a.** IFAN, outside view of the museum; **b.** IFAN, inside view of the museum showing insect boxes; **c.** IITAB, outside view of the museum; **d.** IITAB, inside view of the museum showing insect cabinets and drawers.

The Laboratoire de Zoologie des Invertébrés Terrestres (formerly Section Entomologie) was created in 1945 by André Villiers. It is housed in the Institut Fondamental d’Afrique Noire Cheikh Anta Diop (IFAN), Université Cheikh Anta Diop, Dakar in Senegal (Fig. 1a–b). The IFAN insect collection was established to serve as a centre for entomological collection-based research in West Africa. With over 400,000 specimens, the IFAN insect museum is the largest insect collection in West Africa. Dr. Abdoul Aziz Niang, a specialist of Phlebotomine sandflies (Diptera: Psychodidae), is the current Curator and Director of the IFAN insect collection.

The International Institute of Tropical Agriculture (IITA) is a non-profit international research organisation founded in 1967. Headquartered in Ibadan, Nigeria, IITA is a member of the Consultative Group for International Agricultural Research. IITA has stations and hubs in Central, Eastern, Southern and West Africa (www.iita.org). The IITAB, IITA Biodiversity Center (formerly IITA insect centre or museum) is housed at the Benin Station of IITA in Calavi, Cotonou, Benin (Fig. 1c–d). With over 365,000 specimens, the IITAB collection is the second largest insect collection in West Africa. Dr. Georg Goergen, Entomologist and Biosystematist, is the current Curator and Director of the IITAB.

The taxonomic impediment, which affects biologists around the world, impacts West African researchers especially hard. Most African scientists must rely on distant experts for the identification of insect specimens. Africa produces far fewer trained insect taxonomists than any other continent in the world (de Carvalho et al. 2005). This gap in taxonomic knowledge has also limited the assessment of biodiversity in Africa (Coleman 2015). Currently, the Biodiversity Center of IITA, Benin (IITAB) is one of the major insect identification hubs in Africa.

**Figure 2.**
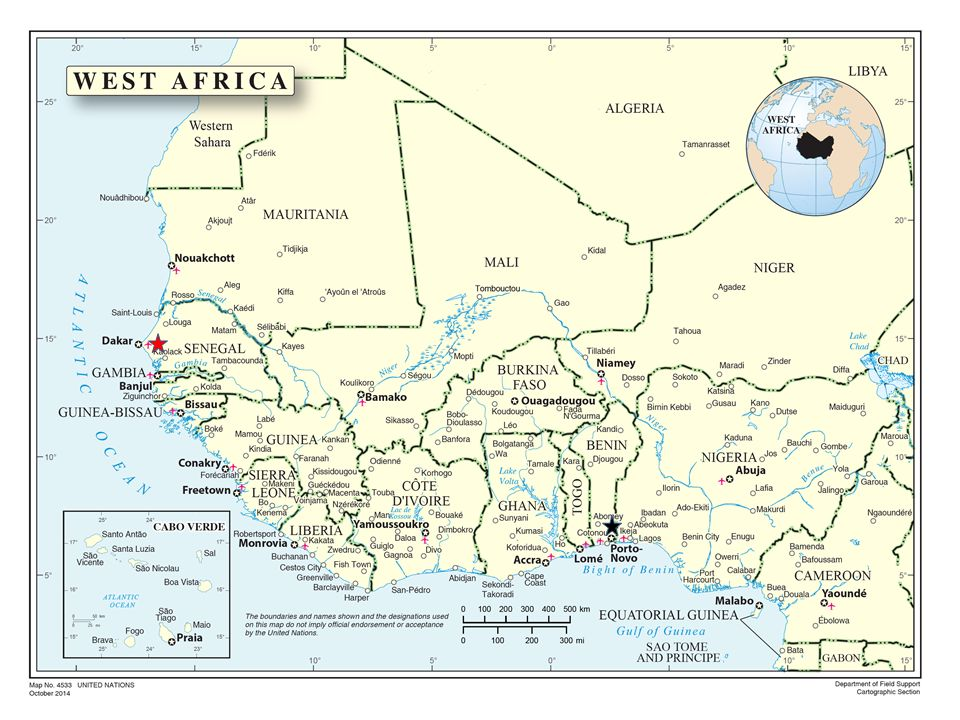
Map of West Africa (United Nations 2014).

The IFAN and IITA insect collections are the two largest in West Africa, a region situated between the Tropic of Cancer and Equator, covering 6,140,000 km^2^, approximately one fifth of Africa. More than 75% of the land consists of plains lying below an elevation of 300 m. The region includes 16 countries: Benin, Burkina Faso, Cape Verde, Côte d’Ivoire, Gambia, Ghana, Guinea, Guinea-Bissau, Liberia, Mali, Mauritania, Niger, Nigeria, Senegal, Sierra Leone and Togo. Additionally, the United Nations recognises Saint Helena (a United Kingdom Overseas Territory), Ascension and Tristan da Cunha as part of West Africa (Comité Inter-états de Lutte contre la Sécheresse dans le Sahel 2016, Kabo-bah and Diji 2018) (Fig. 2).

Given the importance of coccinellids and the patchy knowledge of their diversity in West Africa, we summarised data about the taxonomic holdings of this family in IFAN and IITAB insect collections with the goals of raising the visibility of those institutions and providing a preliminary inventory of West African Coccinellidae (WAC). This work represents part of ongoing research on the WAC by the senior author, who is currently developing a formal taxonomic catalogue of West African Coccinellidae.

## Materials and methods

Museum specimens were studied at the IFAN and IITAB insect collections in West Africa. We photographed specimens and recorded label data (e.g. taxonomic determination, collection locality, collection date, associated plants, collector, determiner etc.).

The taxonomic names that were recorded on museum specimens were put in a database and updated to currently valid names using the systematics literature. Aberrations, variations and subspecies were not included in the database except when museum specimens were identified as such. References are provided, when appropriate, to clarify the current classification for species. In some cases, specimen determinations were made (by KH) using the literature along with examination of reference collections of authoritatively identified material at the Musée Royal de l'Afrique Centrale (MRAC), Museum für Naturkunde der Humboldt-Universität (ZMHB), Museum of Comparative Zoology (MCZ), National Museum of Natural History (NMNH) and University of Georgia Collection of Arthropods (UGCA). Annotations are given when an invalid determination on a specimen label has been updated to the currently valid taxonomic name.

Many museums and institutions kindly assisted this study by hosting visits, providing data or initiating loans of specimens for this and related studies. These collections and institutions include the following:

**CERAAS**, Centre d’Etude Régional pour l’Amélioration de l’Adaptation à la Sécheresse, Université Cheikh Anta Diop de Dakar, Senegal.

**IFAN**, Institut Fondamental d’Afrique Noire Cheikh Anta Diop, Dakar, Senegal.

**IITAB**, International Institute of Tropical Agriculture, Benin Station, Cotonou, Benin.

**MCZ**, Museum of Comparative Zoology, Harvard University, Cambridge, MA, U.S.A.

**MRAC**, Musée Royal de l'Afrique Centrale, Tervuren, Belgium.

**NMNH**, National Museum of Natural History (formerly USNM: United States National Museum), Washington D.C., U.S.A.

**UCAD**, Université Cheikh Anta Diop de Dakar, Dakar, Senegal.

**UEM/IPD**, Unité d'Entomologie Médicale, Institut Pasteur de Dakar, Dakar, Senegal.

**UGA**, University of Georgia, Athens GA, U.S.A.

**UGCA**, University of Georgia Collection of Arthropods, Georgia Museum of Natural History, Athens, GA, U.S.A.

**ZMHB**, Museum für Naturkunde der Humboldt-Universität, Berlin, Germany.

Other abbreviations used in the text:

**DRC**, Democratic Republic of the Congo.

The updated specimen database was used to produce the first list of West African coccinellid diversity (Table 1) and to explore general characteristics about the museum holdings. Valid taxonomic names and tribal placement follow Seago et al. (2011). Generic and species names are arranged alphabetically. The lists of synonyms provided below valid names are not exhaustive, but instead include only junior synonyms that have been used in literature regarding West African taxa or ones that appear on specimen determination labels in the focal collections.

## Checklists

### Checklist of West African Coccinellidae in IFAN and IITAB museums (see Table 1 for more details)

#### Serangium kunowi

Weise, 1892

6C08AE98-8273-5ED6-90CC-F87BBC52F548

##### Distribution

Zambia

#### Brumoides foudrasii

(Mulsant, 1850)

F145D8A6-249B-5FE8-B59E-2F1B70C4CE19

##### Distribution

Benin, Gambia, Guinea, Nigeria, Senegal

#### Chilocorus distigma

(Klug, 1835)

987BB720-000F-53CD-8C9D-D5F81C0C0A0E

##### Distribution

Mozambique

#### Chilocorus dorhni

Mulsant, 1850

611CE9A1-C8DB-5C3A-A1A9-AEFDC24B20DA

##### Distribution

Senegal

#### Chilocorus schioedtei

Mulsant, 1850

5C66671C-194C-51EE-8692-7949F89AEC69

##### Distribution

Benin, Cameroon

#### Chilocorus simoni

Sicard, 1907

E283D897-7362-5CE2-8821-FC62B886D75D

##### Distribution

South Africa

#### Exochomus flavipes

(Thunberg, 1781)

2FBF8BEF-EB91-561E-A288-6E2885FA8404

##### Distribution

Gabon

#### Exochomus laeviusculus

Weise, 1909

7A6E4183-D702-56C1-A936-89A0CD234694

##### Distribution

Benin, Côte d’Ivoire, Guinea, Mali, Mauritania, Senegal, Togo

#### Exochomus nigrifrons

Gerstäcker, 1871

A0B866A5-E30C-5C9D-94E9-7BB968407E2E

##### Distribution

Mali, Senegal

#### Parexochomus nigripennis

(Erichson, 1843)

82867BBC-4337-5664-A99D-98112C1AFBC4

##### Distribution

Mali, Senegal

#### Exochomus pulchellus

Gerstäcker, 1871

79E7BA08-3F1C-5E17-8503-DB5E015151E6

##### Distribution

Gambia, Niger, Rwanda, Senegal

#### Exochomus troberti

Mulsant, 1850

7D78E719-1F19-5868-A5EA-F2E3B07E46CF

##### Distribution

Burkina Faso

#### Aulis annexa

Mulsant, 1850

9D2EAB15-7F2A-50C7-B256-3E49AC7395DA

##### Distribution

Senegal

#### Clitostethus flavotestaceus

Mader, 1955

DBD81906-B263-5595-8373-5D2DFA36624F

##### Distribution

Senegal

#### Nephus flavomaculatus

Fürsch, 1966

5B760B86-9AF4-5EDD-BF4D-B9BBB9A99371

##### Distribution

Benin, Nigeria

#### Nephus vetustus

Weise, 1915

357A9314-78D6-52AD-81D3-032870015377

##### Distribution

Gabon

#### Nephus phenacoccophagus

Fürsch, 1987

A72D8A23-7357-59FE-9037-0E22ACE44349

##### Distribution

Nigeria

#### Nephus kamburovi

Fürsch, 1992

4BA9A0A8-ECD4-5017-B0DB-602EF59323B0

##### Distribution

Malawi

#### Nephus oblongosignatus

Mulsant, 1850

D324C8AD-A466-5D0D-9AF9-71FDDE06243D

##### Distribution

Tanzania

#### Nephus ornatulus

Korschefsky, 1931

7A6DD1B1-6B22-5268-B455-C5D3AE0F3FB8

##### Distribution

DRC

#### Nephus sudanicus

Weise, 1925

3BFEBA83-41A1-55C9-9D2F-B724A8F97EEF

##### Distribution

Mauritania

#### Scymnobius bilucernarius

(Mulsant, 1850)

F4DF6361-FEBD-5CE8-B642-8189CA31C54B

##### Distribution

Mexico

#### Scymnus canariensis

Wollaston, 1864

D8670A86-01D1-5BA1-AAC1-C2B8EC3FE969

##### Distribution

São Tome and Principe, Senegal

#### Scymnus casstroemi

Mulsant, 1850

1B350FE1-0669-5324-A7EB-930E943913AA

##### Distribution

Guinea, Senegal

#### Scymnus gnavus

Weise, 1895

E054571A-7356-56D5-BE4E-6B94C2B09653

##### Distribution

Guinea

#### Scymnus kibonotensis

Weise, 1910

44A344EB-1700-553A-BA65-7BFC9EC9A047

##### Distribution

Côte d’Ivoire, Guinea, Nigeria

#### Scymnus levaillanti

Mulsant, 1850

81A41B21-DE38-5E9E-A631-FB762FFF45DF

##### Distribution

Nigeria, Malawi

#### Scymnus pruinosus

Weise, 1895

E43D7300-A485-50A7-A955-4845D1EA219A

##### Distribution

Zambia

#### Scymnus monroviae

Casey, 1899

2FCF8766-B290-5EE8-8720-162B04FC155C

##### Distribution

Benin, Côte d’Ivoire, Guinea, Niger, Senegal, Togo

#### Scymnus nigrosellatus

Mader, 1950

63025A4B-B79C-5181-82B7-CF554921EDEC

##### Distribution

Zambia

#### Scymnus quadrivittatus

Mulsant, 1850

9738E438-BFE1-51B5-A1C7-67A1F8E4DF58

##### Distribution

Nigeria

#### Scymnus rubiginosus

Mader, 1950

7D91E966-C223-5568-8A88-898FEA4CC113

##### Distribution

Côte d’Ivoire, Guinea, Senegal

#### Scymnus scapuliferus

Mulsant, 1850

D1BBC229-5A5A-5C02-9D47-745AC3692421

##### Distribution

Benin, Côte d’Ivoire, Guinea, Nigeria, Madagascar, Senegal, Togo

#### Scymnus schoutedeni

Mader, 1950

99E145E3-EC00-5746-AAA7-74C9F1D031B2

##### Distribution

Senegal

#### Scymnus senegalensis

Mader, 1955

E492343F-636F-54F2-A535-55D45B8E03BD

##### Distribution

Côte d’Ivoire, Gambia, Guinea, Mali, Mauritania, São Tome and Principe, Senegal

#### Scymnus villiersi

Mader, 1955

3CA780B0-326B-5355-BA7E-44D6A693023D

##### Distribution

Niger, Senegal

#### Stethorus aethiops

Weise, 1899

ACB57C94-B1ED-5044-9C74-1D78DA667457

##### Distribution

Benin, Ghana, Guinea-Bissau, Mozambique

#### Stethorus endroedyi

Fürsch, 1970

4F6EC29C-B255-5DB5-9C5A-B38A91AEF55B

##### Distribution

Malawi

#### Stethorus jejunus

Casey, 1899

0EA19E48-8314-5F7B-8E8B-E797B0C48599

##### Distribution

Ghana, Nigeria, Mozambique

#### Adalia bipunctata

(Linnaeus, 1758)

6AB2552E-C104-5828-998E-01858BDCCFEC

##### Distribution

Cameroon

#### Anisolemnia decempustulata

Weise, 1888

8740EBD4-5BD2-5CFC-9201-EF9F5539BC4A

##### Distribution

Togo

#### Bulaea anceps

(Mulsant, 1850)

F830F859-02F9-5B71-91D5-A402E4D1217A

##### Distribution

Mozambique

#### Caria welwitschii

Crotch, 1874

54F76728-8648-5E70-8053-6340CDCA98A7

##### Distribution

Guinea

#### Cheilomenes aurora

Gerstäcker, 1871

5C30D7D2-C9DD-54D7-BCB8-807AF27F1E69

##### Distribution

Tanzania

#### Cheilomenes lunata

(Fabricius, 1775)

039ACC42-54E5-5D9A-A850-C7A70EBCE416

##### Distribution

Benin, Burkina Faso

#### Cheilomenes sulphurea

(Olivier, 1791)

38306156-309E-5BA4-9296-14E9F6B99C49

##### Distribution

Angola, Cameroon

#### Cheilomenes propinqua

(Mulsant, 1850)

43D175AD-0B9F-500C-8B15-92160DB6C9B3

##### Distribution

Côte d’Ivoire, Gabon

#### Cheilomenes quadrilineata

(Mulsant, 1850)

B08456B4-BCB2-5E03-9998-A16257FCD339

##### Distribution

Senegal

#### Coccinella intermedia

(Crotch, 1874)

B8500504-FED1-5405-8BAE-650EFE34F58D

##### Distribution

São Tome and Principe

#### Coccinella septempunctata

Linnaeus, 1758

11792B51-197D-5D4E-88C3-8A1CD7677D44

##### Distribution

Cape Verde

#### Declivitata hamata

(Thunberg, 1808)

8104F430-F761-5685-B0DA-BE91FF3D2B94

##### Distribution

Senegal

#### Declivitata uncifera

Fürsch, 1967

9AB6D0F0-4055-587B-89A5-333EE7FE78C7

##### Distribution

Cameroon

#### Harmonia vigintiduomaculata

(Fabricius, 1792)

A22708E4-F70C-584C-BAF7-AB0EB9AECB94

##### Distribution

Benin, Liberia, Nigeria, Togo

#### Hippodamia variegata

(Goeze, 1777)

335DC6A9-8C00-5153-B368-131F70AFD80C

##### Distribution

Colombia, Nigeria, Senegal, Tanzania

#### Lemnia machadoi

Mader, 1952

D8C4A968-F1B7-5CBC-8E09-3D5840269877

##### Distribution

Cameroon

#### Megalocaria dilatata

(Fabricius, 1775)

917472C2-D77D-5B71-821A-698C574109B4

##### Distribution

Benin

#### Micraspis lineola

(Fabricius, 1775)

C64F6216-086F-5E31-B90F-90F84ADFCC7A

##### Distribution

Togo

#### Micraspis striata

(Fabricius, 1792)

B32DF781-9B4D-5549-8D88-C504FB949886

##### Distribution

Côte d’Ivoire, Gabon

#### Psyllobora bisoctonotata

(Mulstant, 1850)

CC91E27C-F108-51AC-B82F-7D266005964E

##### Distribution

Senegal

#### Psyllobora lutescens

(Crotch, 1874)

9E9682D5-3020-5A68-B710-5F8F9DE7F939

##### Distribution

Guatemala

#### Psyllobora variegata

(Fabricius, 1781)

036CE85C-635D-56D7-B4A9-2A0DEF6586DA

##### Distribution

South Africa

#### Xanthadalia effusa

(Erichson, 1843)

921AEBB7-F849-57B5-A109-FB7E9CF46487

##### Distribution

Benin, DRC

#### Xanthadalia rufescens

Mulsant, 1850

E376E7EB-580B-562B-B482-66AB41987CAD

##### Distribution

Benin, Mali, Mauritania, Senegal

#### Diomus hennesseyi

Fürsch, 1987

A7DE0649-BBBC-596D-BCAB-018BAAD32D50

##### Distribution

Nigeria

#### Chnootriba elaterii

(Rossi, 1794)

491F19F1-D594-5926-9780-01BB715C3F53

##### Distribution

Benin, Côte d’Ivoire, Gambia, Guinea, Liberia, Mali, Mauritania, Nigeria, São Tome and Principe, Senegal

#### Chnootriba hirta

(Thunberg, 1781)

2AA87447-7A03-5FB5-9A30-2FCC5A71CD1A

##### Distribution

Guinea, Tanzania

#### Chnootriba similis

(Thunberg, 1781)

C9DE0E27-4C4D-5753-9B16-5BC11A21F09D

##### Distribution

Benin, Burkina Faso

#### Cleta punctipennis

(Mulsant, 1850)

7EEC920E-FDA5-502A-9255-AACD0D177DCE

##### Distribution

Togo

#### Cleta sahlbergi

(Mulsant, 1850)

053E7477-493C-50EA-A6F3-AB75BF474A4F

##### Distribution

Côte d’Ivoire, Kenya

#### Epilachna bissexguttata

Weise, 1895

A8FC50D7-2455-5275-A2E5-EC0EB5BA9B3A

##### Distribution

Côte d’Ivoire, DRC

#### Epilachna bomparti

Mulsant, 1850

7DBB54EC-77C6-515A-AC5C-8D676E6E294D

##### Distribution

Liberia, Senegal

#### Epilachna colorata

Mulsant, 1850

462C8061-1250-5D39-A245-B2A973EC8EF8

##### Distribution

Cameroon

#### Epilachna iocosa

(Mader, 1941)

169A3C8E-B305-528B-AB91-C49F2E6EAA5C

##### Distribution

South Africa

#### Epilachna nigritarsis

Mulsant, 1850

5B67661B-0EF8-594A-ABCC-FC704A942111

##### Distribution

Cameroon

#### Epilachna vigintipunctata

Mulsant, 1850

A68D6FAC-0F18-58E7-94F6-75C3167514D3

##### Distribution

Liberia, Tanzania

#### Henosepilachna atropos

(Sicard, 1912)

846C306C-466E-5593-BFFA-5AEB9B66C8C7

##### Distribution

Equatorial Guinea

#### Henosepilachna bisseptemnotata

(Mulsant, 1853)

7498AF38-2BFF-51C4-B1F2-579CC58DC36B

##### Distribution

Tanzania

#### Henosepilachna clavareaui

(Weise, 1901)

3E22E012-F4C3-54E2-A68A-67148C5BA9B9

##### Distribution

Benin

#### Henosepilachna ertli

(Weise, 1906)

5311A71F-2F85-54DF-8655-261A24D2A557

##### Distribution

Côte d’Ivoire, Liberia

#### Henosepilachna fulvosignata

(Reiche, 1847)

BAEFAE7F-C8D1-555A-A0FE-FBEC61AC0C5C

##### Distribution

Côte d’Ivoire

#### Henosepilachna moseri

(Weise, 1903)

CB309483-53C2-5A92-8435-760FC9FC9233

##### Distribution

Equatorial Guinea

#### Henosepilachna reticulata

(Olivier, 1791)

D62E1FD2-D258-5916-9EF7-CF6392EA5AE5

##### Distribution

Benin, Mali, Niger, Nigeria, Senegal

#### Henosepilachna simplex

(Weise, 1895)

62A58A88-ABD5-538B-B541-73DA01945900

##### Distribution

Liberia

#### Solanophila canina

(Fabricius, 1781)

8B2CA986-4056-5CD1-99E1-C95064859686

##### Distribution

Guinea

#### Solanophila dregei

(Mulsant, 1850)

7F669D7A-815E-59FA-8089-C8E3F350AFE2

##### Distribution

Côte d’Ivoire

#### Solanophila scalaris

(Gerstäcker, 1871)

6DE54F2B-3C49-53B2-AA36-5A177259740A

##### Distribution

Tanzania

#### Hyperaspis aestimabilis

Mader, 1955

80C4E12E-B249-57BD-AA30-EE2211E4D6B5

##### Distribution

Angola, DRC

#### Hyperaspis centralis

Mulsant, 1850

475B8890-4115-511A-AB08-2665D8EFF7F5

##### Distribution

Mexico

#### Hyperaspis delicatula

(Mulsant, 1850)

E8896E7D-4093-5429-9D43-577243C41EE8

##### Distribution

Benin, Gambia, Ghana, Guinea-Bissau, Nigeria, Malawi

#### Hyperaspis lugubris

(Randall, 1838)

C5DD9997-6D2F-52C6-AFE0-5BF69639E608

##### Distribution

Ghana, Nigeria

#### Hyperaspis maindroni

(Sicard, 1929)

FFB7EE79-1179-56DC-939C-03827DAA19F6

##### Distribution

Mauritania, Niger, Senegal

#### Hyperaspis merckii

(Mulsant, 1850)

6D4A602C-4D50-5731-A1B2-A04B4B382279

##### Distribution

Mauritania, Senegal

#### Hyperaspis pumila

Mulsant, 1850

EEE926FA-E163-5A5E-B24A-8E187F04874F

##### Distribution

Gambia, Guinea, Guinea-Bissau, Niger, Nigeria, Senegal, Togo

#### Hyperaspis senegalensis

(Mulsant, 1850)

C2267008-C788-552B-8DA3-52EB950FE9DA

##### Distribution

Gambia, Ghana, Nigeria, Senegal, Sierra Leone, Malawi

#### Hyperaspis sericea

Fürsch, 1972

4007B2E6-E527-5021-B877-DA72F665FABC

##### Distribution

Malawi

#### Hyperaspis vinciguerra

Capra, 1929

FA4C8459-72D9-5A29-8E62-ED272E16E384

##### Distribution

Gambia, Senegal, Malawi

#### Tenuisvalvae notata

(Mulsant, 1850)

A8D79B2C-99B8-5003-87EE-440F982C3128

##### Distribution

Benin, Bolivia

#### Ortalia ovulum

Weise, 1898

75D4E5F3-20DA-557A-A8B5-CCE07B685A8C

##### Distribution

Liberia, Mali, Togo

#### Rodolia cardinalis

(Mulsant, 1850)

53162E98-068F-5245-8F30-FFBB6C292FA8

##### Distribution

Kenya

#### Rodolia iceryae

Janson in Ormerod, 1887

8B18A899-5B4C-5D0C-9388-9CC83F7AE0EE

##### Distribution

Senegal

#### Rodolia occidentalis

Weise, 1898

0436B09A-DA2A-591D-9C96-114C54EFBD50

##### Distribution

Benin, Ghana, Nigeria, Senegal

#### Rodolia senegalensis

Weise, 1913

837B3D0F-00B4-5CBE-970A-C9F92DD91DA8

##### Distribution

Senegal

#### Platynaspis capicola

Crotch, 1874

01612FAC-08A3-59B6-AAFD-488C1968F5F0

##### Distribution

DRC

#### Platynaspis ferruginea

Weise, 1895

59D78062-FFAA-545D-A636-942A48B43856

##### Distribution

Benin, Togo

#### Platynaspis kollari

Mulsant, 1850

5D3B29FC-524D-5FDF-AFB5-A8BF1385A688

##### Distribution

Liberia

#### Platynaspis obscura

Gorham, 1901

7A2101B4-7A6D-5847-952D-56F0B403E16A

##### Distribution

Côte d’Ivoire, Liberia

#### Platynaspis pilosa

Sicard, 1930

2B97EBBA-2DFC-5408-805D-1C8A61FE1EB5

##### Distribution

South Africa

#### Platynaspis rufipennis

Gerstäcker, 1871

F05691A6-54E1-5457-B8F3-FE32316291F4

##### Distribution

Côte d’Ivoire, Liberia, Niger

#### Platynaspis vittigera

Weise, 1895

027F9CEB-94FF-5737-A613-16DA63A24284

##### Distribution

DRC

#### Pharoscymnus sexguttatus

(Gyllenhall, 1808)

3357EFFA-9D97-513E-A7ED-3E536B63A400

##### Distribution

Ghana

#### “Leis” “maculata”

F9412911-A954-57F8-9927-21B56604D202

##### Distribution

Côte d’Ivoire

## Analysis

The taxonomically updated list of coccinellid species, present in the IFAN and IITAB collections, includes 129 species, representing 40 genera assigned to 11 tribes and two subfamilies following the classification of Seago et al. (2011). A total of 751 West African coccinellid specimens was recorded for the two collections. Of those, 385 specimens (68 spp., 30 genera) are deposited at IITA, while 366 specimens (84 spp., 31 genera) are at IFAN Table 1.

Most specimens (62%) were curated under currently valid names; however, 38% of specimens were labelled using junior synonyms. At IITA, 83% of the specimens were labelled using currently valid species names, while at IFAN, 39% of specimens were labelled using valid names.

**Figure 3.**
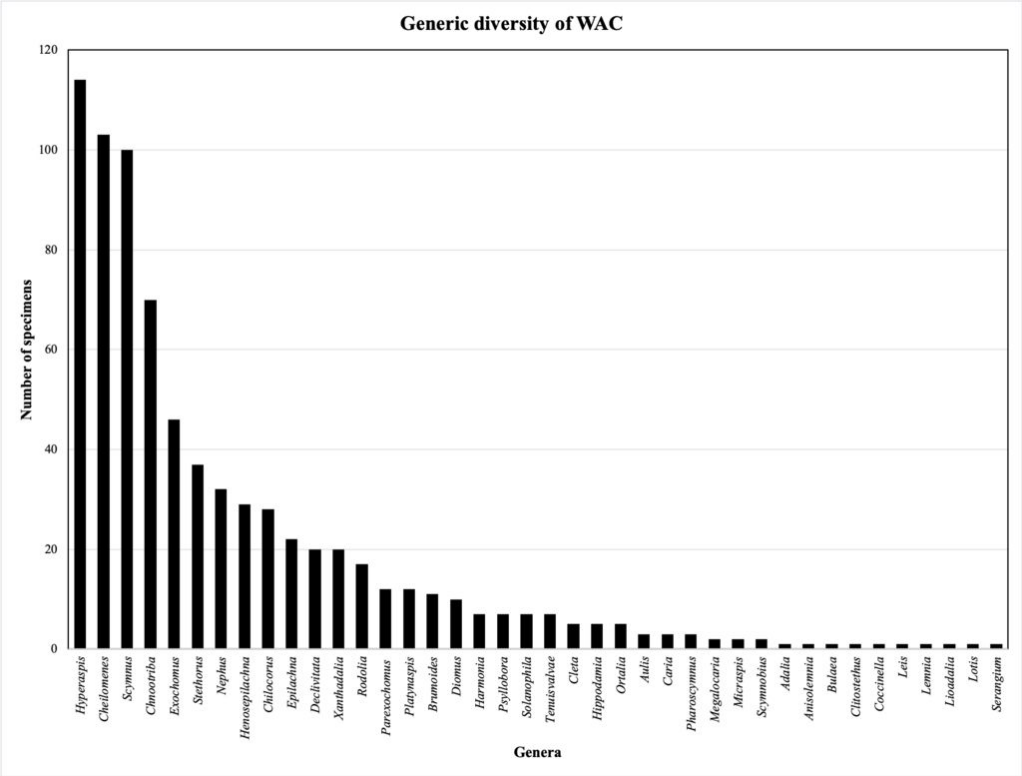
Generic-level specimen representation in the IFAN and IITAB Collections.

Five genera comprise 57% of the specimens: *Exochomus* (6%), *Chnootriba* (9%), *Scymnus* (13%), *Cheilomenes* (14%) and *Hyperaspis* (15%) (Fig. 3). Twenty-five (of 40) genera each represent less than 1% of the total specimens. The remaining genera (*Chilocorus*, *Declivitata*, *Epilachna*, *Henosepilachna*, *Nephus*, *Platynaspis*, *Rodolia*, *Stethorus* and *Xanthadalia*) each account for between 2 and 5% of the overall specimen total.

*Cheilomenes lunata* (Fabricius, 1775), *Cheilomenes propinqua* (Mulsant, 1850), *Cheilomenes sulphurea* (Olivier, 1791), *Chnootriba elaterii* (Rossi, 1794), *Chnootriba similis* (Thunberg, 1781), *Exochomus laeviusculus* Weise, 1909, *Hyperaspis delicatula* (Mulsant, 1850) and *Hyperaspis pumila* Mulsant, 1850, are the most abundant species in the collections (Fig. 6). Specimens identified as *Scymnus* sp. make up the third most numerous group appearing in these collections.

### Geographic distribution

The coccinellid holdings in these two collections originated in 35 countries with 85% of specimens coming from West African countries, 14% coming from other African countries (DRC, Gabon, Madagascar, Malawi, Mozambique, Rwanda, Tanzania and Zambia) and 1% from non-African countries. More than half (66%) of West African material, housed in these two collections, came from just five countries: Senegal (27%), Nigeria (26%), Benin (5%), Liberia (4%) and Côte d'Ivoire (4%).

**Figure 4.**
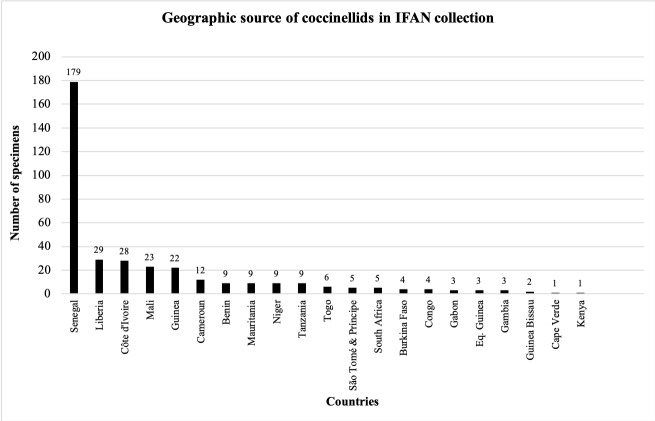
Geographic source of coccinellid specimens in the IFAN Collection.

**Figure 5.**
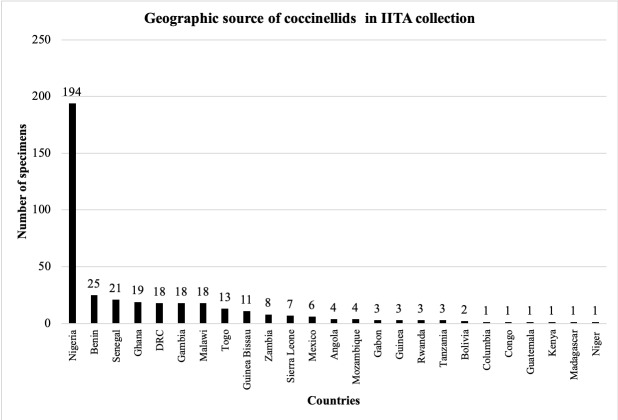
Geographic source of coccinellid specimens in the IITAB Collection.

**Figure 6.**
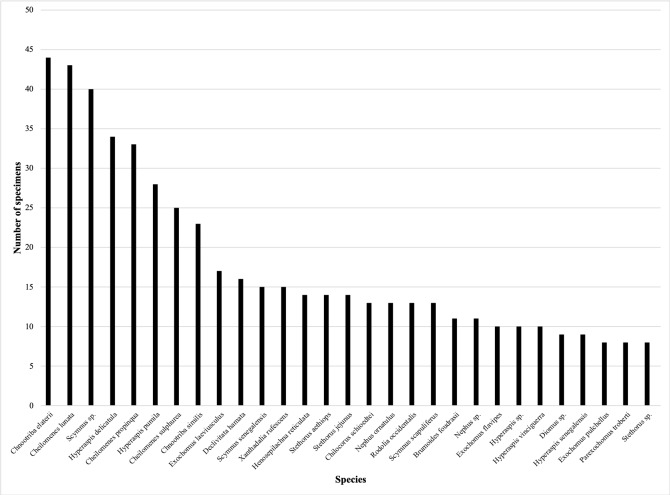
Specific-level specimen representation of coccinellid holdings of the IFAN and IITAB Collections.

West African specimens housed in the IFAN museum were collected from 22 African countries. Most of these specimens (77%) were from five countries: Senegal (49%), Liberia (8%), Côte d’Ivoire (8%), Guinea (6%) and Mali (6%) (Fig. 4). The coccinellid specimens in the IITA originated in 25 countries, including 10 non-West African countries and four non-African countries (Bolivia, Columbia, Guatemala and Mexico). Most lady beetle specimens in the IITA museum (72%) were collected from five West African countries: Nigeria (50%), Benin (6%), Gambia (5%), Ghana (5%) and Senegal (5%). Two non-West African countries were represented: DRC (5%) and Malawi (5%) (Fig. 5).

### Temporal distribution

**Figure 7.**
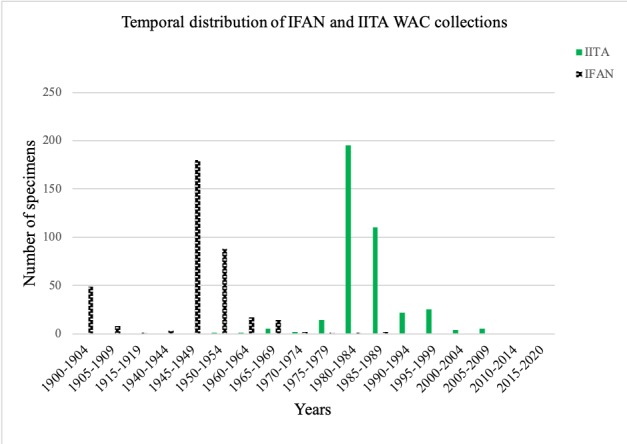
Temporal distribution of IFAN and IITAB collection of coccinellids.

Coccinellid material in the IFAN and IITA insect collections differ in temporal coverage (IFAN: 1900–1994; IITAB: 1950–2009) (Fig. 7). In the IFAN collection, the oldest specimens were all collected in 1900 and represent the following species: *Adalia bipunctata* (Linnaeus, 1758), *Anisolemnia decempustulata* Weise, 1888, *Aulis annexa* Mulsant, 1850, *Brumoides foudrasii* (Mulsant, 1850), *Caria welwitschii* Crotch, 1874, *Cheilomenes aurora* Gerstäcker 1871, *Cheilomenes lunata* (Fabricius, 1775) and *Chnootriba elaterii* (Rossi, 1794). A single specimen of *Stethorus aethiops* Weise, 1899, collected in 1950, represents the oldest coccinellid record in the IITAB collection.

Both collections show a spike in growth of coccinellid holdings during one decade, but not the same one (Fig. 7). While 17% of the IFAN WAC specimens were collected between 1900 and 1944, the great majority (73%) were collected between 1945 and 1954. Only 5% of the specimens were added between 1955 and 1994. No new coccinellid material has been added since 1994.

The IITAB WAC records indicate that 6% of specimens were collected between 1950 and 1979, 80% from 1980 to 1989 and 14% between 1990 and 2009. No new coccinellid material was added after 2009.

## Discussion

Data records compiled from collection labels in the IFAN and IITAB insect collections show that both collections combined provide an historical record of West African coccinellid diversity spanning over a century. It is clear that much coccinellid diversity in this region remains unrecorded though.

Very little published information is available about African coccinellids. Fürsch (1992) reports 70 species from Western Uganda. In Algeria, 75 species were recorded (Lakhal et al. 2018). The West African region, with its surface area of 6,140,000 km^2^, is nearly 26 times the size of Uganda (236,040 km^2^) and more than twice the size of Algeria.

West Africa, with its diverse ecosystems, landscapes, bioclimatic regions and vegetation (desert, rain forest, savannah), should support one of the highest diversities of coccinellids in all of Africa. The current total of 129 known coccinellid species from West Africa is surprisingly low for such a heterogeneous region.

The two focal collections of this study, the largest biodiversity centres in West Africa, differ in their taxonomic coverage. The IFAN holds more West African coccinellid diversity (31 gen., 84 spp.) than IITA (30 gen., 68 spp.). One possible explanation for the higher taxonomic diversity at IFAN is that their coccinellid records span nearly a century (1900–1994) while records at IITA only range from 1950 to 2009. In addition, the holdings at IFAN were enhanced by many expeditions to other West African countries, especially Côte d’Ivoire, Liberia, Mali, Mauritania, Senegal and Togo (Paulian et al. 1983).

A species name can become invalid due to the discovery of an older valid name or due to subsequent reclassification of the species in a different genus. Even though there was more taxonomic diversity represented at IFAN, 61% of species names used in the collection have not been updated to the valid names used in the current classification. At IITAB, however, most coccinellid species names (83%) were current and valid. It should also be noted that the various researchers, who have served as curators of IFAN insect collection, were taxonomists. Even though their expeditions and fieldwork focused on insect biodiversity in general, their efforts were concentrated on their respective specialities. These researchers each left Africa after some time and were no longer involved in the curation of these collections (e.g. André Villiers: 1945–1956, Michel Condamin: 1950–1973 and 1978–1988; Roger Roy: 1958–1992, Bernadette Soltani: 1988, Aïssatou Dramé: 1988–1991, Sun Heat Han: 1992–1996) (A. Niang pers. comm.). These are some of the potential reasons why the taxonomy of the coccinellid holdings at these museums was not current.

The IITA arthropod collection plays a crucial taxonomic role by providing essential, authoritative insect identifications amongst other services (e.g. biodiversity monitoring, pest management control etc.). IITA research has contributed to the description of more than 120 arthropod species (Ortiz 2017). This position of the IITA helps to explain why the identifications of its WAC specimens are more current: the IITAB WAC collection is newer than the one at IFAN.

Considering the numbers of specimens, *Exochomus* (6%), *Chnootriba* (9%), *Scymnus* (13%), *Cheilomenes* (14%) and *Hyperaspis* (15%) are the most strongly represented West African genera in the two collections. *Cheilomenes lunata*, *Cheilomenes propinqua*, *Cheilomenes sulphurea*, *Chnootriba elaterii* and *Chnootriba similis* are the most commonly collected species. Whereas *Cheilomenes lunata*, *C. propinqua* and *C. sulphurea* are widespread aphid predators, *Chnootriba elaterii* and *Ch. similis* are serious herbivorous pests of major staple crops. All these species may have been collected more often because they are relatively large, more colourful than many other coccinellids in the region and are regularly occurring on many cultivated and wild plants. In contrast, the collection, preparation and identification of tiny, brown coccinellids, like *Scymnus* species, are more difficult and time consuming. Drab, minute coccinellids could have been abundantly collected in field samples, but might never have been prepared, identified and curated. As a result, these less conspicuous coccinellids could be greatly under-represented in museum holdings even though they might be very common and important in various agroecosystems.

Many predaceous species are represented in the holdings, such as *Exochomus flavipes* (Thunberg, 1781), *Exochomus laeviusculus* Weise, 1909, *Stethorus jejunus* Casey, 1899, *Hyperaspis delicatula*, *H. pumila*, *Rodolia cardinalis* (Mulsant, 1850) and *Scymnus senegalensis* Weise, 1913. Some of these species are poorly represented in these collections, but this is likely due to collection and preparation biases, rather than actual rarity in the region. Although these relative abundance numbers of specimens in the collections are not the result of systematic and long-term sampling efforts, the simple spatial and temporal records of occurrence for these species in the region provide important information that could facilitate entomological research and pest management programmes in the sub-region.

Records of material in both IFAN and IITAB show that more than 60% of WAC specimens were collected from five countries (Senegal, Nigeria, Benin, Liberia and Côte d'Ivoire). These countries might have experienced more collecting effort because they either house the museums (Benin and Senegal) or because they are neighbouring countries where museum expeditions could be easily conducted (Côte d'Ivoire, Liberia, Nigeria). Benin, Côte d’Ivoire, Nigeria and Senegal have been agricultural research and trade centres in West Africa since the colonial period (Paulian et al. 1983). IFAN (Senegal), IITA (Benin, Nigeria) and ORSTOM (Côte d’Ivoire) were originally established to promote scientific research before the 1950s. Studies conducted through those organisations have continued to be published in more recent years (Chazeau and Couturier 1985, Fürsch 1991a, Sæthre et al. 2011), generating specimens for the museums.

The collecting efforts that built these museum holdings were haphazard, not the result of long-term, systematic monitoring efforts in the region. More than 23% of IITAB WAC specimens were collected from the IITA Station in Ibadan, Nigeria, while 17% of IFAN WAC were collected in Dakar, Senegal. Despite the high historical value of both collections, the geographic record is uneven. Some countries were far more heavily sampled over the years than others.

The coccinellid material housed in both collections is diverse, but there is a surprising lack of overlap in taxa between the two collections, even though they are in neighbouring countries that have similar ecological habitats. At IFAN, there are 56 WAC species that are not present in IITAB. There are 35 WAC species represented in the IITAB holdings that are not found in the IFAN collection. The lack of overlap could be due to collecting biases in the projects or expeditions that occurred at each institution. A large percentage of taxa represented in these museums was collected by only a few individuals (Fig. 3) which is consistent with that scenario. In fact, the combined WAC diversity at these two collections (40 genera, 129 species), represents only 75% of genera and 50% of the species already known to occur in the region as reported in the literature (KH). This lack of overlap between collections may be due in part from being at an early stage of discovery and collection development for WAC diversity. It is clear that there is a need for much more thorough study and sampling in order to accurately assess the diversity of this important group across West Africa.

It is noteworthy that both collections include African coccinellids from outside West Africa. The IITAB also has material from Central and South America (Bolivia, Columbia, Guatemala and Mexico). These non-West African specimens were probably received as exchanges between international collaborators who were conducting general systematics research or were collaborating with the various Insect Pest Management programmes carried out by IITA. These collaborations with researchers from around the world might help to explain why the identifications of material at IITAB were taxonomically more current than at IFAN. In fact, most of the IITAB coccinellids were identified by a German researcher, Helmut Fürsch, a taxonomic authority of Afrotropical Coccinellidae.

The IFAN and IITA WAC collections each show a history that is marked by three distinct periods of development. For IFAN, these significant periods occurred in 1900-1944, 1945-1954 and 1955-1994. IFAN holds some material that predates the establishment of the insect collection in the 1940s. This older material (collected 1900-1944) came from many European collections and collectors (e.g. P. Daget, E. Fleutiaux, M. Griaule, T. Jackson, Delattre, H. Junod, A. Vuillet etc.). In 1945, André Villiers established the IFAN entomology section and began to organise many expeditions to African locations, including Cameroon (1939, 1951), Senegal (1945–1956), Casamance, Senegal (1946), Mali (1946, 1947), Guinea (1946, 1954), Côte-d'Ivoire (1946, 1955), Guinea Bissau (1947), Aïr Mountains, Niger (1947), Senegalese Ferlo (1948, 1950), Mauritania (1948–1953), southern Nigeria (1949), Benin and Togo (1950), southern Togo (1950), Sudan region (border of Senegal with South of Sahara), Fernando-Poo Island, Equatorial Guinea (1951) and Niokolo-Koba National Park, Senegal (1955, 1956). These field trips and Villiers’ collaborations with a network of foreign entomologists resulted in a decade (1945–1954) during which most of IFAN WAC specimens (73% of the total WAC holdings) were collected. The period of rapid growth of WAC holdings at IFAN ceased after Villiers returned to the Muséum National d'Histoire Naturelle (Paris) in 1956. Although he organised many subsequent trips to Africa between 1961 and 1977, that was a period of great change in the region. In the 1960s, most West African countries became independent and experienced major transitions and restructuring of administration.

The noteworthy periods of development for the IITAB WAC collection were 1950–1979, 1980–1989 and 1990–2009. Not surprisingly, IFAN’s slower growth in the 1960s coincided with the lowest rate of growth for IITA’s new collection. In the late 1970s, however, there were major pest outbreaks (e.g. Maize streak virus, cassava mealybug, cassava green mite, mango mealybug, fruit tree mealybug etc.) that led IITA scientists to establish collaborative integrated pest management programmes with Central and South American researchers at the International Center for Tropical Agriculture (CIAT) (Agounké et al. 1988, Ortiz 2017). These biological control programmes were very successful in importing, rearing and releasing natural enemies to manage these pests (Agounké et al. 1988, Ortiz 2017). During research trials for those projects, insects were sampled from agricultural lands. In fact, more than 40% of WAC specimens in the IITA museum were collected between 1979 and 1989 on cassava alone. It is likely that many coccinellids in the IITA collection were collected because the 1970s pest outbreaks pushed the institution to establish partnerships with stakeholders, international researchers and other African research institutions. From 1990 to the present, records from the IITA museum show a very significant decrease in the number of coccinellids collected. This decreased rate of growth could be explained by the spectacular success of the biological pest control programme carried out by IITA on cassava, mango trees and other crops, thereby reducing the need for field sampling. In addition to the identified material referred to in the present paper, there is still a huge backlog of unidentified coccinellid specimens at IITAB. The non-WAC specimens obtained at that same time are another benefit from those same events and resulting collaborations.

Despite the success of IPM programmes and taxonomic expeditions led by IFAN and IITA in many African countries, it is clear that some groups of West African coccinellid genera with high known diversity were poorly sampled (e.g. species of *Adalia*, *Anisolemnia*, *Clitostethus*, *Coccinella*, *Diomus*, *Megalocaria*, *Micraspis*, *Nephus*, *Psyllobora*, *Rodolia*, *Scymnus* etc.). For example, 22 species of *Nephus* have been reported to occur in West Africa (KH), yet only 6 are represented in these collections.

This gap in taxonomic knowledge about lady beetles mirrors the situation seen in many other insect taxa in West Africa. The assessment of biodiversity in the region has been hampered historically by a lack of local taxonomic expertise, inaccessibility of scientific literature, rarity of reliable arthropod reference collections, limited scientific infrastructure and a lack of financial resources. Recent advances in systematics, especially in “cybertaxonomy,” now provide web-based taxonomic tools, diverse publication outlets and easy access to a wealth of digitised scientific resources including technical literature, high quality photographs, specimen data etc., thereby reducing the taxonomic impediment for researchers in places like West Africa. If coupled with strategic development of international, institutional collaborations to conduct biodiversity surveys and inventory projects, great progress could be made towards filling large taxonomic and geographical gaps in our knowledge of West African insects.

## Figures and Tables

**Table 1.** Updated list of coccinellid holdings of the IFAN and IITAB collections, where: a*: present at IFAN, but not from West African locality, b*: present at IITAB, but not from West Africa locality, **a**: present at IFAN, West African source, **b**: present at IITAB, West African source and **ab**: in both museums. The current list is limited to specific rank only. Subspecies and aberrations are mentioned only if they were found on specimen determination labels.

**Taxon**	**Collection localities**	**References**
**Family Coccinellidae Latreille, 1807**		
**Subfamily Microweiseinae Leng, 1920**		
**Tribe Serangiini Pope, 1962**		
*Serangium kunowi* Weise, 1892*^1^	Zambia^b*^	Escalona and Ślipiński 2012
**Subfamily Coccinellinae Latreille, 1807**		
**Tribe Chilocorini Mulsant, 1846**		
*Brumoides foudrasii* (Mulsant, 1850)	Benin^b^, Gambia^b^, Guinea^b^, Nigeria^b^, Senegal^a^	Mader 1954
= *Exochomus foudrasii* Mulsant, 1850		
*Chilocorus distigma* (Klug, 1835)	Mozambique^b*^, Nigeria^b^, Senegal^a^	
*Chilocorus dorhni* Mulsant, 1850	Senegal^a^	
*Chilocorus schioedtei* Mulsant, 1850	Benin^ab^, Cameroon^a*^, Ghana^b*^, Guinea^a^, Nigeria^b^, Senegal^a^	Chazeau and Couturier 1985, Samways et al. 1999
= *Chilocorus discoideus* Crotch, 1874		
*Chilocorus simoni* Sicard, 1907	South Africa^ab*^	
*Exochomus flavipes* (Thunberg, 1781)	Gabon^b^, Madagascar^b*^, Nigeria^b^, Senegal^a^	
*Exochomus laeviusculus* Weise, 1909	Benin^a^, Côte d’Ivoire^a^, Guinea^a^, Mali^a^, Mauritania^a^, Senegal^a^, Togo^a^	
*Exochomus nigrifrons* Gerstäcker, 1871*^2^	Mali^a^, Senegal^ab^	Biranvand et al. 2017a, Łączyński and Tomaszewska 2012
= *Brumus nigrifrons* Gerstäcker, 1871		
= *Brumus fulviventris* Fairmaire, 1884		
= *Brumus trivittatus* Weise, 1891		
= *Brumus nigeriana* Korschefsky, 1938		
= *Brumus nigrifrons nigerianus* Korschefsky, 1938		
*Parexochomus nigripennis* (Erichson, 1843)	Mali^a^, Senegal^a^	Biranvand et al. 2017b, Li et al. 2016, Kovář 2007
= *Exochomus nigripennis* (Erichson, 1843)		
= *Exochomus nigromaculatus nigripennis* Crotch, 1874		
*Exochomus pulchellus* Gerstäcker, 1871	Gambia^b^, Niger^a^, Rwanda^b*^, Senegal^a^	
*Exochomus troberti* Mulsant, 1850	Burkina Faso^a^, Nigeria^b^, Senegal^a^	Hodek et al. 2012, Mohamed et al. 2018, Kovář 2007
= *Exochomus flavipes troberti* Mulsant, 1850		
**Tribe Coccidulini Mulsant, 1846**		
= **Scymnini Mulsant, 1846**		
= **Exoplectrini Crotch, 1874**		
*Aulis annexa* Mulsant, 1850	Senegal^a^	
*Clitostethus flavotestaceus* Mader, 1955	Senegal^a^	
*Nephus flavomaculatus* Fürsch, 1966	Benin^b^, Nigeria^b^	
*Nephus vetustus* Weise, 1915	Gabon^b^, Nigeria^b^	
*Nephus phenacoccophagus* Fürsch, 1987	Nigeria^b^	
*Nephus kamburovi* Fürsch, 1992	Malawi^b*^	
*Nephus oblongosignatus* Mulsant, 1850	Tanzania^b*^	
*Nephus ornatulus* Korschefsky, 1931	DRC^b*^, Ghana^b^, Nigeria^b^, Rwanda^*^, Senegal^b^, Sierra Leone^b^, Togo^b^	Fürsch 1966
= *Scymnus ornatulus* Korschefsky, 1931		
*Nephus sudanicus* Weise, 1925	Mauritania^a^	
*Scymnobius bilucernarius* (Mulsant, 1850)	Mexico^b*^	Gordon and González 2002
= *Nephus bilucernarius* (Mulsant, 1850)		
*Scymnus canariensis* Wollaston, 1864	São Tome and Principe^a*^, Senegal^a^	
*Scymnus casstroemi* Mulsant, 1850	Guinea^a^, Senegal^a^	
*Scymnus gnavus* Weise, 1895	Guinea^a^	
*Scymnus kibonotensis* Weise, 1910	Côte d’Ivoire^a^, Guinea^a^, Nigeria^b^	
*Scymnus levaillanti* Mulsant, 1850	Nigeria^b^, Malawi^b*^	
*Scymnus pruinosus* Weise, 1895	Zambia^b*^	
*Scymnus monroviae* Casey, 1899	Benin^a^, Côte d’Ivoire^a^, Guinea^a^, Niger^a^, Senegal^a^, Togo^a^	
*Scymnus nigrosellatus* Mader, 1950	Zambia^b*^	
*Scymnus quadrivittatus* Mulsant, 1850	Nigeria^b^	
*Scymnus rubiginosus* Mader, 1950	Côte d’Ivoire^a^, Guinea^a^, Senegal^a^	
*Scymnus scapuliferus* Mulsant, 1850	Benin^a^, Côte d’Ivoire^a^, Guinea^b^, Nigeria^b^, Madagascar^b*^, Senegal^a^, Togo^a^	
*Scymnus schoutedeni* Mader, 1950	Senegal^a^	
*Scymnus senegalensis* Mader, 1955	Côte d’Ivoire^a^, Gambia^a^, Guinea^a^, Mali^a^, Mauritania^a^, São Tome and Principe^a^, Senegal^a^	
*Scymnus villiersi* Mader, 1955	Niger^a^, Senegal^a^	
*Stethorus aethiops* Weise, 1899	Benin^b^, Ghana^b^, Guinea-Bissau^b^, Mozambique^b*^, Nigeria^b^, Sierra Leone^b^, Tanzania^b*^	
*Stethorus endroedyi* Fürsch, 1970	Malawi^b*^	
*Stethorus jejunus* Casey, 1899	Ghana^b^, Nigeria^b^, Mozambique^b*^, Tanzania^b*^	
**Tribe Coccinellini Latreille, 1807**		
*Adalia bipunctata* (Linnaeus, 1758)	Cameroon^a*^	
*Anisolemnia decempustulata* Weise, 1888	Togo^a^	Chazeau and Couturier 1985, Mader 1954
= *Anisolemnia 10-pustulata* Weise, 1888		
*Bulaea anceps* (Mulsant, 1850)	Mozambique^b*^	Fürsch 2005
= *Isora circularis* Mader, 1941		
*Caria welwitschii* Crotch, 1874	Guinea^a^	
*Cheilomenes aurora* Gerstäcker 1871	Tanzania^a*^	Fürsch 1975
= *Cydonia aurora* Gerstäcker, 1871		
*Cheilomenes lunata* (Fabricius, 1775)	Benin^a^, Burkina Faso^a^, Cameroon^a*^, Côte d’Ivoire^a^, Gabon^a*^, Gambia^a^, Guinea^a^, Guinea-Bissau^a^, Liberia^a^, Mali^a^, Senegal^a^, West Africa^a^, South Africa^a*^, Tanzania^a*^	Fürsch 1991a
= *Cydonia lunata* (Fabricius, 1775)		
= *Cydonia lunata vulpina* (Fabricius, 1798)		
= *Cydonia lunata vulpiphursa* Olivier, 1791		
*Cheilomenes sulphurea* (Olivier, 1791)	Angola^b*^, Cameroon^a*^, Côte d’Ivoire^a^, Democratic Republic of Congo (DRC)^b*^, Gabon^b*^, Ghana^b^, Nigeria^b^, Malawi^b*^, Rwanda^b*^, Senegal^a^	Eizaguirre 2007
= *Cheilomenes orbicularis* Casey, 1899		
= *Cheilomenes sulphurea sulphurea* (Olivier, 1791)		
*Cheilomenes propinqua* (Mulsant, 1850)	Côte d’Ivoire, Gabon^b*^ , Guinea-Bissau^ab^, Mali^a^, Mauritania^a^, Niger^a^, Nigeria^b^, Senegal^ab^	Eizaguirre 2007, Fürsch 1979
= *Cheilomenes vicina* (Mulsant, 1850)		
= *Cydonia vicina* Mulsant, 1850		
= *Cheilomenes vicina vicina* Mulsant, 1850		
=*Cheilomenes propinqua vicina* Mulsant, 1850		
*Cheilomenes quadrilineata* (Mulsant, 1850)	Senegal^a^	
= *Cydonia 4-lineata* Mulsant, 1850		
*Coccinella intermedia* (Crotch, 1874)	São Tome and Principe ^a*^	Gordon 1987a, Nematollahi 2016
= *Lioadalia intermedia* Crotch, 1874*^3^		
= "*Cydonia intermedia*" Cramer		
*Coccinella septempunctata* Linnaeus, 1758	Cape Verde^a^	
*Declivitata hamata* (Thunberg, 1808)	Senegal^a^	Fürsch 1987
= *Alesia hamata* (Mulsant, 1850)		
= *Micraspis striata* (Crotch, 1874)		
= *Alesia striata* (Gemminger & Harold, 1876)		
= *Alesia striata hamata* (Weise, 1898)		
= *Declivitata hamata* Fürsch, 1964		
*Declivitata uncifera* Fürsch, 1967	Cameroon^a*^, DRC^b*^, Guinea-Bissau^b^	
*Harmonia vigintiduomaculata* (Fabricius, 1792)	Benin^b^, Liberia^a^, Nigeria^b^, Togo^b^	Coutanceau 2008
= *Stictoleis vigintiduomaculata* (Fabricius, 1792)		
= *Stictoleis 22-maculata* (Fabricius, 1792)		
*Hippodamia variegata* (Goeze, 1777)	Colombia^b*^, Nigeria^b^, Senegal^b^, Tanzania^a*^	GBIF Secretariat 2019, Gordon 1987b
= *Adonia variegata* (Goeze, 1777)		
*Lemnia machadoi* Mader, 1952	Cameroon^a^	Fürsch 2002
= *Dysis sicardi* Mader, 1954		
*Megalocaria dilatata* (Fabricius, 1775)	Benin^b^	Hodek et al. 2012, Poorani 2002
= *Anisolemnia dilatata* (Fabricius, 1775)		
*Micraspis lineola* (Fabricius, 1775)	Togo^b^	Nattier et al. 2015, Ślipiński 2007
= *Alesia lineola* (Fabricius, 1775)		
*Micraspis striata* (Fabricius, 1792)	Côte d’Ivoire^a^, Gabon^b*^, Guinea^a^, São Tome and Principe^a^, Senegal^a*^	Kovář 2007, Ślipiński 2007
= *Alesia striata* (Fabricius, 1792)		
*Psyllobora bisoctonotata* (Mulstant, 1850)	Senegal^a^	Ali et al. 2018
*Psyllobora lutescens* (Crotch, 1874)	Guatemala^b*^	
*Psyllobora variegata* (Fabricius, 1781)	South Africa^a*^	Nicolas 2013
= *Thea variegata* (Fabricius, 1781)		
*Xanthadalia effusa* (Erichson, 1843)	Benin^b^, DRC^a^	
*Xanthadalia rufescens* Mulsant, 1850	Benin^b^, Mali^a^, Mauritania^a^, Senegal^a^	Fürsch 1987
**Tribe Diomini Gordon, 1999**		
*Diomus hennesseyi* Fürsch, 1987	Nigeria^b^	
**Tribe Epilachnini Mulsant, 1846**		
*Chnootriba elaterii* (Rossi, 1794)	Benin^a^, Côte d’Ivoire^a^, Gambia^a^, Guinea^a^, Liberia^a^, Mali^a^, Mauritania^a^, Nigeria^b^, São Tome and Principe^a*^, Senegal^a^	Hodek et al. 2012, Jadwiszczak and Węgrzynowicz 2003, Tomaszewska and Szawaryn 2016
= *Henosepilachna elaterii* (Rossi, 1794)		
= *Epilachna chrysomelina* (Fabricius, 1775)		
= *Epilachna chrysomelina manca* Mader, 1929		
= *Henosepilachna elaterii voltaensis senegalensis* Fürsch, 1964		
*Chnootriba hirta* (Thunberg, 1781)	Guinea^a^, Tanzania^a*^	Tomaszewska and Szawaryn 2016
= *Henosepilachna hirta* (Thunberg, 1781)		
= *Epilachna hirta* (Thunberg, 1781)		
*Chnootriba similis* (Thunberg, 1781)	Benin^a^, Burkina Faso^a^, Côte d’Ivoire^a^, DRC^a*^, Guinea^a^, Liberia^a^, Nigeria^b^, Senegal^a^	Chazeau and Couturier 1985, Jadwiszczak and Węgrzynowicz 2003, Tomaszewska and Szawaryn 2016
= *Chnootriba assimilis* Mulsant, 1850		
= Chnootriba similis ab. repanda Sicard, 1930		
*Cleta punctipennis* (Mulsant, 1850)	Togo^ab^	Tomaszewska and Szawaryn 2016
= *Epilachna punctipennis* Mulsant, 1850		
*Cleta sahlbergi* (Mulsant, 1850)	Côte d’Ivoire^a^, Kenya^a*^	Szawaryn et al. 2015, Tomaszewska and Szawaryn 2016
= *Solanophila sahlbergi* Mulsant, 1850		
*Epilachna bissexguttata* Weise, 1895	Côte d’Ivoire^a^, DRC^a*^, Mali^a^, Niger^a^, Senegal^a^	Jadwiszczak and Węgrzynowicz 2003
= *Epilachna monticola* Weise, 1899		
= *Solanophila monticola* Weise, 1898		
*Epilachna bomparti* Mulsant, 1850	Liberia^a^, Senegal^a^	
*Epilachna colorata* Mulsant, 1850	Cameroon^a*^, Liberia^a^	Jadwiszczak and Węgrzynowicz 2003
= *Epilachna subsignata* Mulsant, 1895		
= *Solanophila subsignata* Mulsant, 1895		
= *Solanophila elliptica* Weise, 1912		
*Epilachna iocosa* (Mader, 1941)	South Africa^a*^	Jadwiszczak and Węgrzynowicz 2003
= *Solanophila 20-punctata* Mader, 1941		
*Epilachna nigritarsis* Mulsant, 1850	Cameroon^a*^, Liberia^a^	Jadwiszczak and Węgrzynowicz 2003
= *Epilachna impatiens* Fürsch, 1960		
*Epilachna vigintipunctata* Mulsant, 1850	Liberia^a^, Tanzania^a*^, Togo^a^	Jadwiszczak and Węgrzynowicz 2003
= *Epilachna punctipennis multinotata* Gerstäcker, 1873		
*Henosepilachna atropos* (Sicard, 1912)	Equatorial Guinea^a*^, Senegal^a^	Jadwiszczak and Węgrzynowicz 2003
= *Epilachna atropos* Sicard, 1912		
*Henosepilachna bisseptemnotata* (Mulsant, 1853)	Tanzania^a*^	Jadwiszczak and Węgrzynowicz 2003
= *Epilachna bisseptemnotata* Mulsant, 1853		
*Henosepilachna clavareaui* (Weise, 1901)	Benin^a^	Fürsch 1991b
= *Epilachna clavareaui* Weise, 1901		
*Henosepilachna ertli* (Weise, 1906)	Côte d’Ivoire^a^, Liberia^a^	Jadwiszczak and Węgrzynowicz 2003
= *Epilachna ertli* Weise, 1906		
*Henosepilachna fulvosignata* (Reiche, 1847)	Côte d’Ivoire^a^	Fürsch 1991b
*Henosepilachna moseri* (Weise, 1903)	Equatorial Guinea^a*^	Jadwiszczak and Węgrzynowicz 2003
= *Epilachna moseri* Weise, 1903		
*Henosepilachna reticulata* (Olivier 1791)	Benin^b^, Mali^a^, Niger^b^, Nigeria^b^, Senegal^a^	Jadwiszczak and Węgrzynowicz 2003, Heinrichs and Barrion 2004
= *Epilachna reticulata* (Olivier 1791)		
*Henosepilachna simplex* (Weise, 1895)	Liberia^a^	Jadwiszczak and Węgrzynowicz 2003
= *Epilachna simplex* Weise, 1895		
*Solanophila canina* (Fabricius, 1781)	Guinea^a^	
*Solanophila dregei* (Mulsant, 1850)	Côte d’Ivoire^a^	Tomaszewska and Szawaryn 2016
= *Epilachna dregei* Mulsant, 1850		
*Solanophila scalaris* (Gerstäcker, 1871)	Tanzania^a*^	Mader 1941
= *Epilachna scalaris* (Gerstäcker, 1871)		
**Tribe Hyperaspini Mulsant, 1846**		
*Hyperaspis aestimabilis* Mader, 1955	Angola^b*^, DRC^b*^, Malawi^b*^, Zambia^b*^	
*Hyperaspis centralis* Mulsant, 1850	Mexico	
*Hyperaspis delicatula* (Mulsant, 1850)	Benin^b^, Gambia^b^, Ghana^b^, Guinea-Bissau^b^, Nigeria^b^, Malawi^b*^, Sénégal^b^, Sierra Leone^b^, Togo^b^	
*Hyperaspis lugubris* (Randall, 1838)	Ghana^b^, Nigeria^b^	Gordon 1985
= *Hyperaspis jucunda* LeConte, 1852		
*Hyperaspis maindroni* (Sicard, 1929)	Mauritania^a^, Niger^a^, Senegal^a^	Biranvand et al. 2017b, Ali et al. 2018
*Hyperaspis merckii* (Mulsant, 1850)	Mauritania^a^, Senegal^a^	
*Hyperaspis pumila* Mulsant, 1850	Gambia^b^, Guinea^b^, Guinea-Bissau^b^, Niger^a^, Nigeria^b^, Senegal^b^, Togo^b^	Biranvand et al. 2017b
*Hyperaspis senegalensis* (Mulsant, 1850)	Gambia^b^, Ghana^b^, Nigeria^b^, Senegal^b^, Sierra Leone^b^, Malawi^b*^	
*Hyperaspis sericea* Fürsch, 1972	Malawi^b*^	
*Hyperaspis vinciguerra* Capra, 1929	Gambia^b^, Senegal^b^, Malawi^b*^	
*Tenuisvalvae notata* (Mulsant, 1850)	Benin^b^, Bolivia^b*^, Nigeria^b^	Gordon and Canepari 2008
= *Hyperaspis notata* Crotch, 1874)		
**Tribe Ortaliini Mulsant, 1850**		
*Ortalia ovulum* Weise, 1898	Liberia^a^, Mali^a^, Togo^b^	
**Tribe Noviini Mulsant, 1846**		
*Rodolia cardinalis* (Mulsant, 1850)	Kenya^b*^	
*Rodolia iceryae* Janson in Ormerod, 1887	Senegal^a^	Hounkpati et al. 2019
= *Rodolia iceryae* Janson, 1887		
= *Rodolia obscura* Weise, 1898		
*Rodolia occidentalis* Weise, 1898	Benin^a^, Ghana^a^, Nigeria^a^, Senegal^ab^	
*Rodolia senegalensis* Weise, 1913	Senegal^a^	
**Tribe Platynaspini Mulsant, 1846**		
*Platynaspis capicola* Crotch, 1874	DRC^b*^, Malawi^b*^	
*Platynaspis ferruginea* Weise, 1895	Benin^b^, Togo^b^	
*Platynaspis kollari* Mulsant, 1850	Liberia^a^	
*Platynaspis obscura* Gorham, 1901	Côte d’Ivoire^a^, Liberia^a^	
*Platynaspis pilosa* Sicard, 1930	South Africa^a*^	
*Platynaspis rufipennis* Gerstäcker, 1871	Côte d’Ivoire^a^, Liberia^a^, Niger^a^	
*Platynaspis vittigera* Weise, 1895	DRC^b*^	
**Tribe Sticholotidini Weise, 1901**		
*Pharoscymnus sexguttatus* (Gyllenhall, 1808)	Ghana^b^	
***Nomen nudum***		
“*Leis maculata*”*^4^	Côte d’Ivoire^a^	

## Footnotes

*1 This record is based on two specimens collected by R. Borowka on 12 AUG 1992 Citrus mussel scale in Chipata, Zambia. They were originally identified as “*Chilocorus serangium* Kunowi”.

*2 This was treated as subspecies of *Exochomus nigrifrons* Gerstäcker, 1871 by Mader (1954: 88) (*Brumus nigrifrons nigerianus* Korschefsky). *Brumus nigrifrons nigerianus* was misspelled. Species of the genus *Brumus* Mulsant, 1850 were transferred to *Exochomus* Redtenbacher, 1843.

*3 This record is based on a single specimen determined as “*Cydonia intermedia* Cramer” by Mader. The holotype for this species is from Abyssinia, now Ethiopia.

*4 This is probably a *nomen nudum*.
